# Host‐dependent variability in amyloid‐beta propagation and deposition

**DOI:** 10.1002/alz70855_105111

**Published:** 2025-12-24

**Authors:** Salvatore Saieva, Nazaret Gamez, Yumeng Huang, Claudia Duran‐Aniotz, Javiera Bravo‐Alegria, Catalina Valdes, Rodrigo Morales

**Affiliations:** ^1^ University of Texas Health Science Center at Houston, Houston, TX, USA; ^2^ Houston Methodist Research Institute, Houston, TX, USA; ^3^ The University of Texas Health Science Center at Houston, Houston, TX, USA; ^4^ University of Texas Medical Branch, Galveston, TX, USA; ^5^ Latin American Brain Health Institute (BrainLat), Universidad Adolfo Ibañez, Santiago, Chile; ^6^ The University of Texas Health Science Center at Houston, Houston, TX, USA, Houston, TX, USA

## Abstract

**Background:**

Alzheimer's disease (AD) is the most common form of dementia, characterized by progressive loss of memory and cognitive decline. From a pathological point of view, AD is characterized by the deposition of misfolded the amyloid‐beta (Aβ) and tau proteins in aggregates known as amyloid plaques and neurofibrillary tangles, respectively. Growing evidence highlights heterogenous clinical manifestations and pathological features among AD individuals, and this variability is attributed to the presence of distinct conformational strains of Aβ. Compelling data show that Aβ spreads in a manner akin to infectious prions and, noteworthy, other prion‐like characteristics have been described for Aβ aggregates, such as the capability to accelerate brain pathology upon administration in susceptible mice, the potential inter‐individual transmission (although under rather uncommon conditions), and the acquisition of different conformational variants, namely the strains.

**Methods:**

In this study, we investigated, by means of immunostaining and Thioflavin S (ThS) staining, the differences in amyloid propagation and deposition upon intracerebral delivery of human and mouse brain homogenate into different animal models (APP/PS1 or Tg2576 mouse models) of amyloid pathology.

**Results:**

Our histological evaluations show that the same inoculum induced different patterns of Aβ pathology in cortex and hippocampus of Tg2576 and APP/PS1 mice: in particular the human‐derived inoculum triggered higher Aβ deposition in APP/PS1 cortex compared to Tg2576 mice. Interestingly, in both cortex and hippocampus of Tg2576 mice, the Aβ burden induced by the human inoculum was lower compared to that induced by the mouse‐derived inoculum, while in the hippocampus of APP/PS1 the Aβ burden was not significantly different.

**Conclusions:**

This study shows that different hosts react differently to the same Aβ sources, thus suggesting the formation of different Aβ strains, which in turn may determine diverse pathological features. Investigating the pathological significance of Aβ strains is also relevant in light of recent reports of potential inter‐individual transmission of Aβ. Finally, our investigations may pave the way to personalized diagnostic approaches and treatments.

